# Diabetic parturient - Anaesthetic implications

**DOI:** 10.4103/0019-5049.71028

**Published:** 2010

**Authors:** Nibedita Pani, Shakti Bedanta Mishra, Shovan Kumar Rath

**Affiliations:** Department of Anaesthesiology, SCB Medical College, Cuttack-753 007, India

**Keywords:** Anaesthesia, diabetes mellitus, pregnancy

## Abstract

Pregnancy induces progressive changes in maternal carbohydrate metabolism. As pregnancy advances insulin resistance and diabetogenic stress due to placental hormones necessitate compensatory increase in insulin secretion. When this compensation is inadequate gestational diabetes develops. ‘Gestational diabetes mellitus’ (GDM) is defined as carbohydrate intolerance with onset or recognition during pregnancy. Women diagnosed to have GDM are at increased risk of future diabetes predominantly type 2 DM as are their children. Thus GDM offers an important opportunity for the development, testing and implementation of clinical strategies for diabetes prevention. Timely action taken now in screening all pregnant women for glucose intolerance, achieving euglycaemia in them and ensuring adequate nutrition may prevent in all probability, the vicious cycle of transmitting glucose intolerance from one generation to another. Given that diabetic mothers have proportionately larger babies it is likely that vaginal delivery will be more difficult than in the normal population, with a higher rate of instrumentally assisted delivery, episiotomy and conversion to urgent caesarean section. So an indwelling epidural catheter is a better choice for labour analgesia as well to use, should a caesarean delivery become necessary. Diabetes in pregnancy has potential serious adverse effects for both the mother and the neonate. Standardized multidisciplinary care including anaesthetists should be carried out obsessively throughout pregnancy. Diabetes is the most common endocrine disorder of pregnancy. In pregnancy, it has considerable cost and care demands and is associated with increased risks to the health of the mother and the outcome of the pregnancy. However, with careful and appropriate screening, multidisciplinary management and a motivated patient these risks can be minimized.

## EPIDEMIOLOGY

The prevalence of gestational diabetes mellitus (GDM) in India varied from 3.8 to 21% in different parts of the country, depending on the geographical locations and diagnostic methods used. GDM has been found to be more prevalent in urban areas than in rural areas.[1–8]

## INTERACTION WITH PREGNANCY

### The effect of diabetes mellitus on mother and foetus

Several pathophysiologic processes that affect both mother [Table 1][9] and foetus [Table 2][10] govern the obstetric and anaesthetic management of the diabetic parturient.

**Table 1:** Major complications of diabetes mellitus

Acute	
Diabetic ketoacidosis	
Hyperosmolar hyperglycaemic nonketotic coma	
Hypoglycaemia	
Chronic	
Macrovascular (atherosclerosis)	
Coronary	
Cerebrovascular	
Peripheral vascular	
Microvascular	
Retinopathy	
Nephropathy	
Neuropathy	
Autonomic	
Somatic	

**Table 2:** Foetal complications of maternal diabetes mellitus

During pregnancy and the puerperium
Chronic
Macrosomia/large for gestational age
Shoulder dystocia
Birth injury/trauma
Structural malformations
CNS: anencephaly, encephalocele, meningomyelocele, spina bifida, holoprosencephaly
Cardiac: transposition of great vessels, ventricular septal defect, situs inversus, single ventricle, hypoplastic left ventricle
Skeletal: caudal regression
Renal: agenesis, multicystic dysplasia
Gastrointestinal: anal/rectal atresia, small left colon
Pulmonary: hypoplasia
Acute
Intrauterine/neonatal death
Neonatal respiratory distress syndrome
Neonatal hypoglycaemia
Neonatal hyperbilirubinaemia
After pregnancy
Glucose intolerance
Possible impairment of cognitive development

Placental insufficiency can be influenced by the anaesthetic technique and can directly impact neonatal well being. Placental abnormalities have been observed even in association with mild, well-controlled gestational diabetes.[11] Maternal hyperglycaemia is noticed promptly by the foetus, because glucose rapidly crosses the placenta to reach concentrations approximately equal to those in the mother. The foetus responds by pouring out insulin from its pancreas. Foetal oxygen consumption rises in direct proportion to the insulin level, resulting in falls in both venous and arterial oxygen content. Oxygen supply is unable to keep pace with demand, resulting in a progressive rise in the arteriovenous oxygen difference between the umbilical artery and vein. Thus, through several mechanisms, acute and chronic hyperglycaemia contribute to foetal hypoxia and acidosis.[12]

### Diabetic ketoacidosis

During pregnancy, diabetic ketoacidosis (DKA) occurs most commonly during the second and third trimesters. It is one of the major causes of foetal mortality and morbidity in diabetic parturients. Foetal loss has been reported to be as high as 50%.[13,14] It is associated with the following factors: (1) bacterial infection; (2) omission of insulin doses in the presence of gastroenteritis because of the parturient’s concern about the possibility of an insulin reaction due to anorexia, nausea, and vomiting; (3) pump malfunction in patients receiving continuous subcutaneous insulin infusion therapy and (4) tocolytic therapy with β-sympathomimetic agents, with or without concomitant glucocorticoid therapy (5) decreased caloric intake, (6) poor medical management, (7) patient non-compliance.[15,16]

Classical presentations include anorexia, nausea, vomiting, polyuria, polydipsia, tachycardia and abdominal pain or muscle cramps. If severe, the picture could include Kussmaul hyperventilation, signs of volume depletion (e.g., hypotension and oliguria), lethargy to coma, normal-to-cold body temperature and a fruity odour noticeable in the patient’s breath. Diabetic parturients can develop ketoacidosis with remarkably low blood glucose values (as low as 200 mg/dL). The presence of ketones, maternal arterial pH of less than 7.30, decreased serum bicarbonate level and elevated anion gap confirm the diagnosis.[17]

### Treatment

(1) Intravenous hydration—initial treatment to be given with normal saline at a rate of 15 to 20 mL/kg/h, 400 mL/m^2^/h, or approximately 1 L/h for the first 2 h of the resuscitation. In the third and subsequent hours 7.5 mL/kg/h according to the clinical situation and urine output. 5% glucose in water to be given when the blood glucose level comes down to 300 to 250 mg/dL, (2) intravenous insulin, (3) treatment of underlying cause of DKA, (4) careful monitoring of serum glucose and electrolytes, (5) bicarbonate is to be administered if the maternal pH is less than 7.10, (6) left uterine displacement to be maintained, (7) supplemental oxygen to be given.

Hypoglycaemia is a continuing health threat in diabetic parturient, especially those who receive insulin therapy. Symptomatic awareness of hypoglycaemia and counter regulatory responses may be inadequate in some diabetic parturient with an autonomic neuropathy.[18] β adrenergic receptor blocking agents are to be avoided in diabetic patients.[19] In hospitalized patients with DM major risk factors for hypoglycaemia include renal insufficiency and decreased caloric intake.[20] In one study, two diabetic pregnant patients (who were receiving insulin therapy) became hypoglycaemic while fasting before caesarean section.[21] The elevated progesterone levels of pregnancy are associated with delayed gastric emptying.[22–24] Delayed and unpredictable gastric emptying can make glycaemic control difficult and result in wide swings in postprandial glucose values. Severe hypoglycaemia in the latter half of pregnancy may be associated with a modest degree of foetal bradycardia, as low as 100 beats/min.

Impaired cardiac adjustment - Airaksinen and colleagues made an interesting observation by using echocardiography to assess, in diabetic parturients, the adaptation of the heart to an increase of blood volume during pregnancy.[25] The pregnancy-induced increase in left ventricular size, stroke volume and heart rate were observed to be less in diabetic parturients. These impairments in diabetic parturient, may be due to preclinical diabetic cardiomyopathy and subclinical autonomic neuropathy. Judicious volume expansion and use of epidural anaesthesia for caesarean section might be preferred.

Hypertension and pre-eclampsia are common problems among diabetic gravidas. Severe pre-eclamtic women with nephropathy (White class F)[26] may have low serum albumin levels and very low colloid oncotic pressures in addition to the usual degree of haemodynamic fragility. So they are vulnerable to pulmonary oedema if they are vigorously volume loaded in preparation for regional anaesthesia.

**Figure 1:**
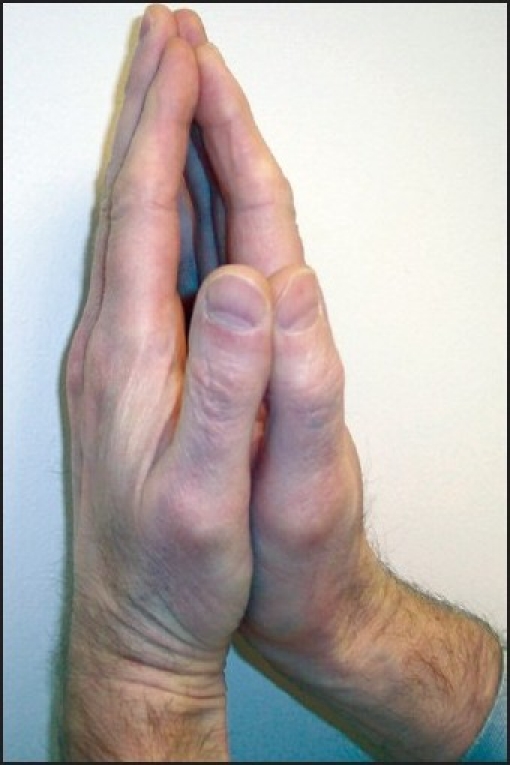
Prayer sign[27] Inability to approximate the palmer surfaces of the phalangeal joints (prayer sign) despite maximal effort, secondary to diabetic stiff-joint syndrome

Stiff joint syndrome is a rare condition consisting of juvenile onset diabetes, nonfamilial short stature and joint contractures. Limited atlanto-occipital extension might make intubation difficult and awake tracheal intubation with or without fiberoptic bronchoscopy may be necessary. A ‘prayer sign’, [Figure 1][27] which is defined by the parturient’s inability to approximate the palmar surfaces of the phalangeal joints despite maximal effort, secondary to diabetic stiff joint syndrome, may be beneficial for the anaesthesiologist to detect patients with associated involvement of the atlantooccipital joint. Other investigators have suggested phalangeal visualization of an ink print of the palm as an alternative screen for difficult intubation.[28] Some authors recommend preanaesthetic flexion--extension radiographic studies of cervical spine, followed by awake intubation in affected patients.[29]

Diabetic scleroderma is synonymous with stiff joint syndrome. There is one case report[30] of a pregnant patient with pregestational DM and diabetic scleroderma who experienced an anterior spinal artery syndrome after administration of epidural anaesthesia for caesarean section. It may be due to (1) pre-existing microvascular disease, (2) an epidural space that may be stiff due to connective tissue disease and (3) administration of a large volume (e.g. 35 mL) of the local anaesthetic agent.

### Gestational diabetes

Pregnancy induces progressive changes in maternal carbohydrate metabolism. As pregnancy advances insulin resistance and diabetogenic stress due to placental hormones necessitate compensatory increase in insulin secretion. When this compensation is inadequate gestational diabetes develops. GDM is defined as carbohydrate intolerance with onset or recognition during pregnancy.[31] Women diagnosed to have GDM are at an increased risk of future diabetes predominantly type 2 DM and increase in the foetal, neonatal morbidity, future development of obesity, and diabetes in the offspring.[32] The most common presentation of gestational diabetes in mothers is antenatal glycosuria. Pregnant women belonging to a high risk ethnic population (e.g. Indians) require *Universal Screening*. This observation emphasizes the need for an appropriate diagnostic tool to diagnose and method to treat GDM and to incorporate them into the local health service arrangements.[33]

### Screening and diagnosis

*‘A one step procedure with a single glycaemic value’*, to diagnose GDM in the community

WHO criteria based on glucose level > 140 mg/dL at 2 h after, 75 g oral glucose load administered irrespective of whether the pregnant woman had anything to eat or not, was able to correctly identify subjects with GDM, as well as woman with normal glucose tolerance[34] which led to clarity in categorizing abnormal glucose tolerance in pregnancy [Table 3].[35]

**Table 3:** Clarity in categorizing abnormal glucose tolerance in pregnancy

2 h plasma glucose	In pregnancy	Outside pregnancy
> 200 mg/dL	Diabetes	Diabetes
> 140 - 199 mg/dL	Gestational diabetes mellitus	Impaired glucose tolerance
120-139 mg/dL*	Gestational glucose Intolerance	-
< 120 mg/dL	Normal	Normal

* Needs follow up. The term IGT should not be used to indicate any glucose intolerance in pregnancy (as this terminology is used outside pregnancy)

## ANAESTHETIC MANAGEMENT

### Management of labour and delivery

Diabetic mothers are at high risk and should be delivered in an appropriate hospital environment with consultant-led obstetric care. Regular blood glucose monitoring and ensuring that the blood glucose is within normal range reduces the risk of neonatal hypoglycaemia. During labour, it is essential to maintain good glycaemic control [Table 4].[36] A parturient scheduled for induction of labour is given approximately one third of her usual morning dose of intermediate acting insulin and no regular insulin. Capillary blood glucose levels are monitored hourly during labour via finger sticks with a reflectance meter on the labour floor. If the blood glucose goes above 120 mg/dL, an iv infusion of regular insulin is used to bring blood glucose level within normal limits. Non-glucose containing solution is to be given in the iv line. A 5% glucose containing solution can be ‘piggybacked’ into the mainline via an infusion pump at 125 mL/h (2-3 mL/kg/h). On the first postpartum day, approximately one half the prepregnancy dose of intermediate acting insulin is given to the patient with regular insulin if required.

**Table 4:** Plasma glucose and insulin iv fluid

Plasma glucose at time of onset of labour	Insulin/IV fluids
< 70 mg/dL	5% DNS - 100 mL/h
90-120 mg/dL	NS - 100 mL/h
120-140 mg/dL	NS -100 mL/h plus 4 units of Reg. insulin added with IV fluid
140-180 mg/dL	NS - 100 mL/h plus 6 units of Reg. insulin added with IV fluid
>180 mg/dL	NS - 100 mL/h plus 8 units of Reg. insulin added with IV fluid

Drip rate: 16 to 20 drops per minute. Maternal capillary blood glucose to be checked by glucometer every 1 h and drip rate adjusted

### Analgesia for labour and vaginal delivery

Moderate pain of early labour can be relieved with small doses of narcotic drugs (e.g. butorphanol or nalbuphine). The main problem with larger doses of systemic medication is maternal and neonatal respiratory depression. *Epidural* analgesia is associated with a few obvious advantages : it reduces maternal endogenous catecholamine release and indirectly will increase placental blood flow; it reduces the maternal lactic acid production and hence foetal acidosis; it provides excellent pain relief during the first stage as well as during the second stage, especially if forceps delivery is necessary; and an indwelling epidural catheter can be used should a caesarean delivery becomes necessary.

At Brigham and woman’s Hospital induction of epidural analgesia is started with 0.25% bupivacaine, and maintenance analgesia is obtained with a continuous infusion of 0.0625% to 0.125% bupivacaine with 2 μg/mL fentanyl (8–10 mL/h). At the Massachusetts General Hospital, the epidural test dose (1.5% lidocaine with 1:200K epinephrine) contributes to the labour analgesia; a T10 sensory level is obtained with 15 mL 0.04% bupivacaine with 1.66 μg/mL fentanyl, and the maintenance analgesia is obtained with the same mixture infused at 15 mL/h.[37]

For forceps delivery, dense perineal anaesthesia is necessary; this can be provided by 8 to 10 mL 3% 2-chloroprocaine or 2% lidocaine with bicarbonate added. In the absence of an indwelling epidural catheter, *spinal* anaesthesia in the sitting position (hyperbaric bupivacaine with dextrose) can provide good perineal anaesthesia. This technique will provide perineal relaxation and hence less chance of birth trauma; this is especially important for delivery of a large baby. It may be prudent to consider placing an epidural catheter at the time of spinal placement (*combined spinal-epidural technique*), which can be used if there is urgent operative delivery.[37]

### Anaesthesia for caesarean section

Preoperative assessment should cover the areas of increased risk in this group (hypertension and pre-eclampsia, sepsis, renal dysfunction) as well as the normal preoperative history, examination and investigations. The evaluation of end organ damage should be done. Diabetic parturient has additional risks associated with autonomic neuropathy (HTN., Orthostatic hypotension, painless MI, decreased HR variability, decreased response to medication-atropine and propranolol, resting tachycardia, neurogenic atonic bladder, decreased cough reflex threshold, increased incidence of obstructive sleep apnoea and gastroparesis). With the potential for hypotension during regional anaesthesia, non-invasive testing of autonomic function may be useful. Preclinical diabetic cardiomyopathy, autonomic neuropathy and low colloid osmotic pressure from renal protein wasting may exacerbate haemodynamic instability and also put the parturient at risk for pulmonary oedema in the setting of overzealous hydration.

Optimization of glycaemic control is vital over the perioperative period and the use of a short-acting insulin via infusion, titrated against frequent (hourly) plasma glucose measurement, is mandatory in all but the most mild diet-controlled parturients. Intraoperative blood glucose can be monitored and accordingly insulin is infused as per sliding scale [Table 5].[38]

**Table 5:** Intraoperative insulin regimen

Blood glucose level (mmol/L)	Rate/hour (units)
0-3	0 U, call doctor immediately
3.1-6	1 U
6.1-9	2 U
9.1-12	3 U
12.1-15	4 U, repeat after 30 min, call doctor if rising
More than 15	6 U, call doctor immediately

Fluid infusion: 10% dextrose (1 L) with 20 mmol potassium. Insulin infusion: 50 U of actrapid in 50 mL of 0.9% sodium chloride in a syringe driver (This may need to be modified in insulin resistant type 2 DM, i.e. those needing over 100 U of insulin per day.)

General anaesthesia :- Diabetic parturients undergo general anaesthesia either because of operative urgency or other factors that preclude regional anaesthesia. GA can be problematic because of gastroparesis, limited atlanto-occipital joint extension, increased haemodynamic[39] response to intubation, and impaired counter regulatory hormone responses to hypoglycaemia during sleep.[40] Aspiration prophylaxis should consist of a non-particulate antacid immediately before surgery. Metoclopramide (10 mg) can be administered 30-40 min before to enhance forward gastric emptying and to increase lower oesophageal sphincter tone. Histamine-2-receptor antagonist will be given in addition to this. Stiff joint syndrome may affect the airway and atlanto-occipital joint, making intubation difficult. Medications and equipment needed for managing a difficult airway, including those for fiberoptic intubation, should be readily available. Vohra *et al*.[39] observed variation in response to intubation like heart rates, mean arterial pressure and vascular resistance in the diabetic group. Cardiovascular response to hypoglycaemia may be blunted under GA while in RA patients are able to verbalize sensations that may be consistent with hypoglycaemia. Half hourly blood glucose monitoring for patients undergoing GA is recommended.

Regional anaesthesia is positively indicated compared to general and there is no specific concern related to the spinal over the epidural group. Either spinal or epidural anaesthesia may be appropriate for diabetic parturient provided maternal glycaemic control is satisfactory, the patient receives aggressive preanaesthetic volume expansion with a non-dextrose containing balanced salt solution and hypotension is treated aggressively with ephedrine. In severe diabetics epidural anaesthesia may be preferred because of the slower onset of sympathetic blockade.

Spinal anaesthesia can be obtained with 12 mg hyperbaric bupivacaine (0.75%) with 10 μg fentanyl added. Epidural anaesthesia can be obtained with 15 to 25 mL 2% lidocaine with 1:200K epinephrine and 50 μg fentanyl added. For an urgent caesarean section where foetal acidosis is assumed and an indwelling epidural catheter is present, 3% 2-chloroprocaine is the anaesthetic of choice.[37]

Haemodynamic alterations are more common in regional anaesthesia as there is a higher sympathetic blockade and sympathetic tone might also be abnormal in long standing diabetic patients and aortocaval compression by the gravid uterus accentuates hypotension. Proper perioperative positioning and padding of extremities are important to avoid peripheral nerve injuries. No published data are available on CNS infection after regional anaesthesia in diabetic parturients. But DM is a risk factor for the occurrence of spontaneous epidural abscess in non-pregnant patients. So a strict aseptic technique during administration of regional anaesthesia in diabetic parturient should be taken care.

Postoperative analgesia is important for controlling pain and avoiding catecholamine and glucose swings after delivery. Morphine offers excellent analgesia for up to 24 h with minimal sedation and occasional pruritus. Morphine can be given intrathecally (0.2 mg) or epidurally (3 mg). Nonsteroidal anti-inflammatory medications can be given for breakthrough discomfort. Pruritus can be treated with small doses of naloxone, or nalbuphine (5 mg intravenously). Patients who have undergone general anaesthesia where neuraxial morphine cannot be given patient-controlled analgesia with intravenous morphine, dilaudid, or demoral can be given.

### Special anaesthetic considerations

Diabetic parturients with severe pre-eclampsia, and diabetic nephropathy with superimposed hypertension require special care. They require invasive monitoring to assess the fluid status and cardiovascular function. They also require pulse oximetry and monitoring of urine output, coagulation status before regional anaesthesia, and the airway assessment to evaluate airway oedema.

### Postnatal management

Women with gestational diabetes are at increased risk of a recurrence in future pregnancies. The risk of progression in later life to type 2 diabetes is also increased in gestational diabetics.[41] Systematic reviews indicate that fasting glucose levels taken for OGTTs in pregnancy are indicative of risk; the higher the level, the greater the risk, although no definite limits could be determined. A high BMI and insulin use in pregnancy are also related to increased risk. Close and continued follow-up[42] is required in all women with gestational diabetes. It is routine for all gestational diabetics to stop pregnancy-related hypoglycaemic therapy[43] immediately post-partum pending further review and assessment.

Parturients who are at a risk for developing oedema often do so after delivery.[44] Invasive monitoring may be useful when continued in the immediate post-partum period. Infection is the important cause of morbidity in diabetic parturient which should be taken care.

### ICU supportive care for diabetic parturient

Medical illness associated with pregnancy (for example DM) or its complications (described in Tables 1 and 2 account for a major portion of the parturients admitted to ICU.

### Pearl

‘Although care of the mother is the primary concern in most circumstances, attention must also be paid to foetal health and well being. Intensive insulin therapy to treat hyperglycaemia in critically ill patients, which may be due to illness-related insulin resistance and not to previously known diabetes, appears to improve morbidity and mortality’.

Altered maternal physiology, presence of foetus and diseases associated with pregnancy make management challenging. Organ systems adapt to optimize foetal and maternal outcome. But certain concepts must be kept in mind while dealing a diabetic parturient in ICU.

- Position: avoid supine position after 20 weeks gestation; right lateral decubitus or Fowler position (head of bed elevated) preferred for immobilized patient.
- Monitoring: foetal heart tones should be part of vital signs; continuous foetal monitoring after 23 week gestation if maternal condition affects cardiopulmonary function.
- Thromboembolism prophylaxis: unfractionated or low molecular weight heparin if not contraindicated; venous compression stockings may be of lesser benefit.
- Nutrition: address early as pregnant women more susceptible to starvation ketosis.
- Imaging studies: ionizing radiation known to be teratogenic; limit radiographs appropriately but do not withhold if results may lead to therapeutic intervention.
- Initiate intravenous infusion of insulin: titrate to blood glucose goal of 80 to 120 mg/dL; use of insulin infusion protocol helpful.
- Consider switching to subcutaneous injections of long-acting insulin once at stable regimen.
- Monitor for and attempt to avoid hypoglycaemia.
- Hyperglycaemic crisis to be taken care which was discussed earlier.

## CONCLUSIONS

Diabetes in pregnancy has potential serious adverse effects for both the mother and the neonate. Standardized multidisciplinary care including anaesthetists should be carried out obsessively throughout pregnancy. The provision of continuous safe care to minimize and prevent potential problems will enable a successful outcome to the pregnancy.
